# FKN Facilitates HK-2 Cell EMT and Tubulointerstitial Lesions via the Wnt/β-Catenin Pathway in a Murine Model of Lupus Nephritis

**DOI:** 10.3389/fimmu.2019.00784

**Published:** 2019-04-30

**Authors:** Dongdong Fu, Soulixay Senouthai, Junjie Wang, Yanwu You

**Affiliations:** Department of Nephrology, Affiliated Hospital of Youjiang Medical University for Nationalities, Baise, China

**Keywords:** fractalkine, epithelial-mesenchymal transition, Wnt/β-catenin, tubulointerstitial lesion, HK-2 cells, murine model

## Abstract

Fractalkine (FKN), also known as chemokine (C-X3-C motif) ligand 1, constitutes an intriguing chemokine with a documented role in the development of numerous inflammatory diseases including autoimmune disease. Specifically, it has been reported that FKN is involved in the disease progression of lupus nephritis (LN). The epithelial-mesenchymal transition (EMT) plays a significant role in the formation of tubulointerstitial lesions (TIL), which are increasingly recognized as a hallmark of tissue fibrogenesis after injury. However, the correlation between FKN and EMT or TIL in LN has not been determined. To investigate the potential role of FKN in EMT and TIL, MRL lymphoproliferation (MRL/lpr) strain mice were treated with an anti-FKN antibody, recombinant-FKN chemokine domain, or isotype antibody. Our results revealed that treatment with the anti-FKN antibody improved EMT, TIL, and renal function in MRL/lpr mice, along with inhibiting activation of the Wnt/β-catenin signaling pathway. In contrast, administration of the recombinant-FKN chemokine domain had the opposite effect. Furthermore, to further explore the roles of FKN in EMT, we assessed the levels of EMT markers in FKN-depleted or overexpressing human proximal tubule epithelial HK-2 cells. Our results provide the first evidence that the E-cadherin level was upregulated, whereas α-SMA and vimentin expression was downregulated in FKN-depleted HK-2 cells. In contrast, overexpression of FKN in HK-2 cells enhanced EMT. In addition, inhibition of the Wnt/β-catenin pathway by XAV939 negated the effect of FKN overexpression, whereas activation of the Wnt/β-catenin pathway by Ang II impaired the effect of the FKN knockout on EMT in HK-2 cells. Together, our data indicate that FKN plays essential roles in the EMT progression and development of TIL in MRL/lpr mice, most likely through activation of the Wnt/β-catenin signaling pathway.

## Introduction

Lupus nephritis (LN) constitutes one of the most important complications of systemic lupus erythematosis (SLE), which is a multisystem autoimmune disease characterized by the deposition of immune complexes (1, 2). Upwards of 60% of patients with SLE develop LN (3, 4), of which renal fibrosis is the main feature. Notably, if not controlled, LN can lead to renal failure and represents a significant cause of death in patients with SLE. Previously, the majority of studies that focused on LN stressed the importance of glomerular injury (5, 6). Recently, however, growing evidence demonstrates that the response of renal tubule lesions plays a vital role in the progression of renal disease (7). Moreover, research has suggested that LN is more highly linked to tubulointerstitial lesions (TIL) than to glomerular fibrosis, and that tubular proteinuria may occur in LN prior to any other marker, such as microalbumunuria (8), suggesting that tubular damage plays a significant role in the progression of LN (9, 10). In particular, the primary target of LN is the renal tubules and interstititum, resulting in decreased renal function (11); therefore, the primary factor in the development of LN comprises severe TIL (12). TIL is present in a higher proportion of patients with SLE; thus, it has been reported that TIL may serve as a single causative risk factor underlying LN pathological changes (13). Consequently, it would be valuable to explore the pathological process of TIL in LN.

The epithelial-mesenchymal transition (EMT) of renal tubular epithelial cells comprises a canonical pathological process that has also been associated with susceptibility to LN (14). The role of EMT during tissue injury leads to organ fibrosis (deposition of collagens, elastin, tenascin, and other matrix). EMT is characterized by the loss of epithelial surface markers and decrease of adhesion ability in renal tubular epithelial cells along with the induction of mesenchymal marker expression (15), which results in TIL followed by the development of LN. During EMT progression, downregulation, or loss of epithelial markers including E-cadherin and cytokeratin is observed, whereas mesenchymal markers such as vimentin, α-SMA, and fibroblast-specific protein increase. Increasing evidence implicates EMT as a vital step in the pathogenesis of LN (16). Alternatively, Liu et al. found that using specific antibody treatment could ameliorate TIL by improving EMT, inflammation, and fibrosis in a murine model of LN (17).

A variety of cell signaling pathways are involved in the regulation of EMT and TIL, including the Nuclear factor-κB (NF-κB), TGF-β, and Wnt/β-catenin signaling pathways (18). The Wnt/β-catenin pathway plays a crucial role in TIL formation (19), as well as serving as an important regulatory factor promoting the development of EMT in human proximal tubule epithelial HK-2 cells (20). In the Wnt/β-catenin pathway, activation of Wnt factor results in an increased level of β-catenin in the cytoplasm, which ultimately activates transcription of the downstream target genes in the nucleus, such as c-Myc and cyclin D1 (21). As an important regulator of EMT, activation of the Wnt/β-catenin pathway depends on numerous proinflammatory factors, such as angiotensin II (Ang II) and transforming growth factor β1 (TGFβ1) (22). It has been reported that an inhibitor of RAS gene expression, ICG-001, blocked activation of the Wnt/β-catenin pathway in renal tissue (23, 24), along with attenuating interstitial myofibroblast activation and inhibiting renal inflammation and fibrosis. The Wnt/β-catenin pathway is commonly aberrantly activated in the renal tissue of a LN murine model and in patients with LN (18); accordingly, inhibiting its activation can strikingly reduce the incidence of EMT and injury in renal lesions (25). These data indicate that the Wnt/β-catenin pathway plays a critical role in the development and progression of renal lesions.

According to different activation modes and immune functions, macrophages can be divided into classically activated M1 type macrophage (CAMsor M1) and alternately activated M2 type macrophage (AAMsor M2) (26). F4/80 is a monoclonal antibody that recognizes a murine macrophage-restricted cell surface glycoprotein and has been extensively used to characterize macrophage populations in a wide range of immunological studies. During acute inflammation, infection or ischemia, CD11b^+^/F4/80^+^/Gr1^hi^/CCR2^+^/CD62L^+^ macrophages that secrete pro-inflammatory cytokines are rapidly recruited to the kidneys. Macrophage-derived chemokine (MDC) is a Th2-type CC-like chemokine, also known as CCL22, which can induce the chemotactic movement of dendritic cells and Th2 cells. CCL22 and receptor CCR4 are specific. After specific binding, the CCR4/CCL22 axis participates in the development of autoimmune diseases. Studies have found that CCL22 is highly expressed in autoimmune diseases, such as in patients with rheumatoid arthritis, psoriatic arthritis and osteoarthritis and in patients with experimental autoimmune encephalomyelitis (27, 28).

Fractalkine (FKN), also known as CX3CL1, constitutes the only member of the CX3C chemokine superfamily that participates in cell adhesion and regulation of cell growth; moreover, it is also involved in inflammatory immune responses (29). FKN represents one of the factors associated with tissue damage and the accumulation of immune cells into the damaged area (30). Accumulated evidence has suggested that FKN plays a significant role in the disease progression of LN (31). Moreover, we have previously demonstrated that FKN expression was increased in LN model mice and HK-2 cells that were stimulated with lipopolysaccharide, whereas the protein expression of FKN was decreased after methylprednisolone therapy (32). Liao et al. reported that anti-CX3CR1 (the receptor of FKN) also can attenuate lupus nephritis in murine model (33).

In turn, Kim et al. (34) and Jung et al. (35) have indicated that Wnt5a is associated with the expression of chemotactic factors in endothelial and neutrophil cells, such as CXCL8, CX3CL1, and CCL2. However, the molecular mechanism of FKN in the LN process, especially in EMT and TIL, was poorly understood. As a chemokine factor, we speculated that FKN may regulate the Wnt/β-catenin signaling pathway to promote the EMT process in TIL. On the other hand, due to the large accumulation of macrophages in renal inflammation, transiently released macrophage-derived factors may be involved in the activation of NF-κB, TGFβ. In this study, we therefore examined whether FKN could stimulate the process of EMT, NF-kB, TGFβ, CCL22, F4/80, inflammation, and tubulointerstitial fibrosis in a murine model of LN. We also determined whether FKN was involved in the EMT process of Wnt/β-catenin-expressing HK-2 cells. Mechanistically, we ascertained, for the first time, whether FKN up-regulated EMT-related gene signatures (e.g., vimentin, α-SMA), and hence, renal tubulointerstitial fibrogenesis, and the role of the Wnt/β-catenin signaling pathway in this process.

## Materials and Methods

### Cell Culture, Stable Infection, and Grouping

HK-2 cells were obtained from the Cell Center of Fudan University (Shanghai, China), and cultured in DMEM/F12 medium (Gibco) with 10% fetal bovine serum (Gibco) at 37°C in a humidified 5% CO_2_ atmosphere. HK-2 cells were infected with lentiviral vector particle-CX3CL1, lentiviral vector particle-CX3CL1-RNAi, and hU6-MCS-Ubiquitin-EGFP-IRES-negative control according to manufacturer protocol (Shanghai Genechem Co., Ltd.) to effect FKN overexpression or knockdown (KD). After 12 h of infection, cells were cultured in fresh complete medium for 48–72 h, FKN protein expression was examined using western blotting. The cells were divided into nine groups as follows: (1) Control group; (2) FKN-KD group; (3) XAV939 group; (4) FKN-KD + XAV939 group; (5) Ex-FKN group; (6) Ex-FKN + XAV939 group; (7) Ang II group; (8) FKN-KD + Ang II group; and (9) Ex-FKN + Ang II group.

### Cell Viability Assay

The effects of Ang II (Lot: A9290, Solarbio) treatment on HK-2 cell viability were evaluated using the Cell Counting Kit-8 colorimetric assay (Lot: KH741, Dojindo). Briefly, HK-2 cells were seeded at a density of 5 × 10^3^ cells/well (in 100 μL culture medium) in a 96-well plate. After treatment with Ang II (10^−9^, 10^−8^, 10^−7^mol/L) or XAV939 (1, 5, 10 μmol/L) for 12, 24, and 48 h, CCK-8 was added to each well, followed by 2 h incubation at 37°C in a 5% CO_2_ incubator. The absorbance was measured using a TriStar LB 941 multimode microplate reader (Berthold Technologies) at 450 nm. Each experiment was performed in triplicate.

### Cells Apoptosis Analysis

Cell apoptosis was detected by the fluorescein isothiocyanate (FITC)-Annexin V/propidium iodide (PI) apoptosis kit (FITC-Annexin V/PI) (BD Biosciences). Each group of cells was cultured for 48 h. Cells were detached with 0.25% trypsin/EDTA, harvested by centrifugation at 300 g for 5 min at 4°C, washed twice with cold phosphate buffered saline, and then resuspended in 1x Binding Buffer at a concentration of 1 × 10^6^ cells/mL and incubated with 5 μL FITC-Annexin V and 5 μL PI at room temperature in the dark for 15 min according to manufacturer instructions. HK-2 apoptosis was determined by flow cytometry using a FACSCanto II (BD Biosciences) within 1 h.

### Animals and Experimental Protocol

Female MRL/MpJ-Fas^lpr^/J (MRL/lpr) mice were purchased from the Better Biotechnology Co., Ltd. and were used at 12 weeks of age. All mice were housed under specific pathogen-free conditions at 22–25°C and kept in an environment of 40–60% relative humidity in the Animal Research Institute of Youjiang Medical University for Nationalities (Baise, Guangxi Province, P.R. China). The study was approved by the Committee of Animal Care and Use of Youjiang Medical University for Nationalities, and all procedures were performed according to the National Institutes of Health Guidelines. All mice were maintained with 12-h light/12-h dark photoperiods with free access to water and food. MRL/lpr mice were randomly divided into four groups, with five mice in each group: (1) normal control group, receiving intraperitoneal (IP) injection of 1 mL of normal saline per day; (2) IgG group: IP injection of Rat IgG2A Isotype Control (Lot: CAO2315081, RD) 5 μg/mL per day; (3) rFKN group: IP injection of Recombinant Mouse CX3CL1/Fractalkine Chemokine Domain (Lot: 458-MF, RD) 100 ng/mL per day; and (4) Anti-FKN group: IP injection of Mouse CX3CL1/Fractalkine Chemokine Domain Antibody (Lot: EMX0217061, RD) 5 μg/mL daily per day. Metabolic cages were used to collect urine from mice in each group for 24 h. Urine protein was measured as described previously (36). Then, anesthetization was performed with IP injections of a xylazine (5 mg kg^−1^) and ketamine (80 mg kg^−1^) mixture to mice placed in a supine position prior to sacrifice after treatment for 7 days. The glomeruli were harvested and maintained at −80°C until use for RNA and protein extraction. Blood urea nitrogen (BUN) and creatinine (Cr) levels were measured to assess renal function as described previously (36).

### Enzyme-Linked Immunosorbent Assay (ELISA)

The MRL/lpr mouse blood samples were harvested from the retro-orbital plexus after anesthetization using heparinized glass capillary tubes (EDTAK_2_) at the end of the experiment. The serum was separated from the blood by centrifugation for 15 min at 1,000 × g and stored at −20°C until use. The concentrations of serum anti-nuclear antibody (ANA), anti-dsDNA antibody, and anti-Sm antibody in the individual subjects were determined using an ELISA kit according to manufacturer instruction (Cusabio Biotech Co., Ltd.). The absorbance was measured using the TriStar LB 941 multimode microplate reader at 450 nm.

### Histopathological and Immunohistochemistry Analysis

To evaluate renal pathologic changes, kidney tissue samples were fixed overnight with 10% formalin in 0.01 mol/L phosphate buffer (pH 7.2), then embedded in paraffin for histopathology. The slide sections (3–4 μm thickness) were stained with periodic-Schiff-methenamine (PASM) according to standard procedures and for examination under a light microscope. The examination of renal pathology was performed in a blinded fashion.

For IHC, formalin-fixed and paraffin embedded renal sections were prepared as described previously (37), then the slides were dewaxed and hydrated, after which the sections were immersed in 3% methanol hydrogen peroxide for 20–30 min, then normal goat serum was applied for 20 min to block endogenous peroxidase. The sections were incubated with individually primary antibodies against FKN (1:200) (Lot: GR18924-38, Abcam), Wnt-4 (1:200) (SC-3762, Santa Cruz Technologies), F4/80 (Lot: GR3250648-1, Abcam, 1:200 dilution), and CCL22 (Cat#: DF7781, Affinity, 1:200 dilution) overnight at 4°C, then incubated with goat polyclonal secondary antibody for 20 min at 37°C. After incubation with Streptavidin-HRP, the signal was developed using a DAB Substrate Kit (zli-9018, ZSGB-Bio Co., Ltd.). To compare the expression levels of FKN and Wnt-4 in renal cells by IHC, staining intensity was evaluated semiquantitatively according to a previous study (38), and fluorescence intensity was scanned and quantified using Image-Pro Plus v5.1 software (Media Cybernetics Co., Ltd.). An intensity score (HIS) was calculated as follows: IHS = A × B, where A is the number of positive cells 0–1% = 0, 1–10% = 1, 10–50% = 2, 50–80% = 3, 80–100% = 4, and B is a positive cell color intensity rating of 0 (negative), 1 (weak positive), 2 (positive), and 3 (strong positive) (38).

### Quantitative RT-PCR

The kidney tissues were collected in RNase-free tubes and total RNA was extracted using TRIzol reagent (Invitrogen) according to manufacturer instruction. For cDNA synthesis, RT was performed from 2 μg of total RNA using the FastKing RT Kit (KR116, Tiangen). The mRNA expression levels of FKN, α-SMA, vimentin, Wnt-4, β-catenin, c-Myc, cyclinD1, P-NF-κB –p65, NF-κB –p65, TGFβ, F4/80, CCL22, and GAPDH were determined using SuperReal PreMix Plus (SYBR Green) (FP205, Tiangen) based on manufacturer instruction for the Sequence Detection system with the following primers, which were synthesized by GeneCopoeia: *FKN* (HQP067519, MQP028162), vimentin (HQP018489, MQP030457), α-SMA (HQP016095, MQP026492), and E-cadherin (HQP054891, MQP028853), with GAPDH used as an internal control. The other primer sequences used are shown in Table 1. The PCR reaction system consisted of SYBR Green Mix, forward and reverse primer, cDNA, and deionized RNAase-free water. PCR was initially denatured at 95°C for 30 s followed by 95°C for 10 s and 65°C for 30 s for 40 cycles, then 81 cycles at 55–95°C for 10 s for melting curve analysis. The comparative gene expression was calculated by the 2^−ΔΔ*Ct*^ method as described previously (39).

**Table 1:** Primers used for real-time PCR analysis.

**Target**	**Primer sequence**
H-Wnt-4	(F) 5′-TGGCTGGGTTTCTGCTACG-3′
	(R) 5′-CCCGGATTTTGGCGTATC-3′
H-β-catenin	(F) 5′-TGGATTGATTCGAAATCTTGCC-3′
	(R) 5′-GAACAAGCAACTGAACTAGTCG-3′
H-c-Myc	(F) 5′-CGACGAGACCTTCATCAAAAAC-3′
	(R) 5′-CTTCTCTGAGACGAGCTTGG-3′
H-cyclin D1	(F) 5′-GTCCTACTTCAAATGTGTGCAG-3′
	(R) 5′-GGGATGGTCTCCTTCATCTTAG-3′
H-GAPDH	(F) 5′-AAGAAGGTGGTGAAGCAGGC-3′
	(R) 5′- ACCACCCTGTTGCTGTAGCC-3′
M-Wnt-4	(F) 5′-TGAAAGGGCACGGAAAGC-3′
	(R) 5′-GGCGACCAGAACAGAGGAG-3′
M-β-catenin	(F) 5′-TTGCTGCTGGTTGGTTGGAAGG-3′
	(R) 5′-CCAAGACATCTCGCAGTGAACTCC-3′
M-c-Myc	(F) 5′-AAATCCTGTACCTCGTCCGATT-3′
	(R) 5′-CCACAGACACCACATVAATTTC-3′
M-cyclin D1	(F) 5′-CGTATCTTACTTCAAGTGCGTG-3′
	(R) 5′-ATGGTCTCCTTCATCTTAGAGG-3′
M-NF-kB	(F) 5′-TAAGCCGTACACAGCCACTG-3′
	(R) 5'-CCAGGTAAATGGCTGCAGAT-3'
M-TGFβ	(F) 5′-GCAACAATTCCTGGCGTTACCTTG-3′
	(R) 5′-CAGCCACTGCCGTACACCTCC-3′
M-CCL22	(F) 5′-TCTCGTCCTTCTTGCTGTGG-3′
	(R) 5′-TGACGGATGTAGTCCTGGCA-3′
M-F4/80	(F) 5′-TTGTTGGTGGCACTGTGACC-3′
	(R) 5′-GACTTCTGCTTTGGCTGGATG-3′
M-GAPDH	(F) 5′- GCCACCCAGAAGACTGTGGAT-3′
	(R) 5′-TGGTCCAGGGTTTCTTACTCC-3′

### Western Blot

Aliquots of total kidney homogenate from individual animals (50 mg/mL) were diluted in RIPA buffer (Beyotime Biotechnology) containing protease inhibitor cocktail (Cwbiotech) (1:99) and incubated on ice for 30 min. Protein concentration was measured using a bicinchoninic acid (BCA) protein assay kit (Beyotime Biotechnology). After being quantified, protein samples were loaded and separated by 10% sodium dodecylsulfate polyacrylamide gel electrophoresis (SDS-PAGE), then transferred to a polyvinylidene fluoride membrane (GE Healthcare). After transfer, 5% bovine serum albumin (in TBST buffer; Beijing Solarbio Science) was used to block the membrane at room temperature for 1 h. Then, the membranes were pre-incubated with the primary antibodies anti-FKN (Lot: GR18924-38, Abcam, 1:1,000 dilution), anti-α-SMA (Lot: GR282976-20, Abcam, 1:1,000 dilution), anti-vimentin (Lot: GR3186827-2, Abcam, 1:1,000 dilution), anti-E-cadherin (Lot: GR3184955-1, Abcam, 1:1,000 dilution), Wnt-4 (SC-3762, SantaCruz, 1:500 dilution), c-Myc (Cat#10828-1-AP, Proteintech, 1:1,000 dilution), anti-cyclin D1 (Lot: GR312543-18, Abcam, 1:1,000 dilution), P-NF-κB –p65 (S536, Cell signaling,1:1,000 dilution), NF-κB –p65 (D14E12, Cell signaling,1:1,000 dilution), TGFβ (Lot: SC-146, SantaCruz, 1:500 dilution), F4/80 (Lot:GR3250648-1, Abcam, 1:1000 dilution), CCL22 (Cat#: DF7781, Affinity, 1:1,000 dilution) and anti-GAPDH (cat#6-004-1-1g, Proteintech, 1:1500 dilution) at 4°C overnight and then incubated with secondary antibody following washing with TBST three times. To determine the effect of Wnt/β-catenin signaling, the pathway agonist Ang II (10^−7^ mol/L) and antagonist XAV939 (10 μmol/L) were used to treat cells For visualization of detected proteins, immunoblots were analyzed using an enhanced chemiluminescence (ECL, Millipore) western blot detection kit and the peroxidase luminescence intensity was measured using the Universal Hood II Molecular Imager GEL System (Bio-Rad).

### Statistical Analysis

Data are presented as the means ± standard deviation. Inter-group comparisons were analyzed by one-way analysis of variance (ANOVA); for parametric data, multi-factor comparisons were analyzed by Multi-way classification ANOVA and for non-parametric data, the F-test for equality of variances and Newman-Keuls test for heterogeneity of variance were used. All analyses were conducted using SPSS 20.0 software. *p* < 0.05 was considered statistically significant. Each experiment was repeated three times in duplicate.

## Results

### Ang II Promotes Viability of HK-2 Cells

**Figure 1:**
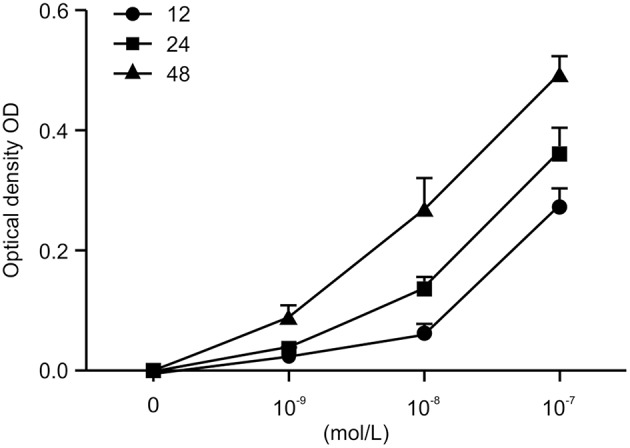
AngII promotes the viability of HK-2 cells. AngII induces dose-dependent increase of cell viability in HK-2 cells. Cells were incubated with indicated concentrations (0, 10^−9^, 10^−8^, 10^−7^mol/L) of AngII for 12, 24, and 48 h. Cell growth promoting activity by AngII was assessed using the CCK-8 assay. Data are expressed as the mean ± standard deviation (*n* = 3). Statistical analyses were performed using one-way ANOVA.

To investigate the role of Ang II on the growth of HK-2 cells, we treated the HK-2 cells with different concentrations of Ang II at various time points (0, 12, 24, and 48 h), then performed the CCK-8 assay to examine cell viability. As shown in Figure 1, after the cells were treated with Ang II at 10^−9^, 10^−8^, or 10^−7^mol/L for the indicated times, the cell viability increased. The half maximal inhibitory concentration (IC50) value for 48 h of Ang II treatment was 10^−7^mol/L (*p* < 0.05). These results show that Ang II could promote HK-2 cell viability in a concentration-and time-dependent manner. On the basis of this observation, we used Ang II at 10^−7^mol/L for 48 h in the following studies.

### FKN Is Involved in HK-2 Cell Apoptosis

**Figure 2:**
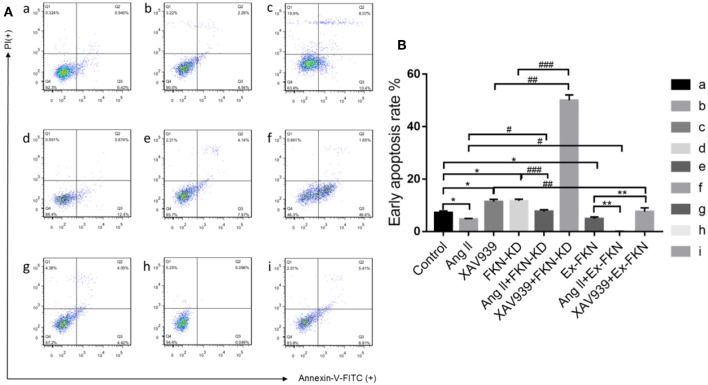
Annexin V-FITC and PI staining to evaluate apoptosis in HK-2 cells following different treatment. The HK-2 cells were divided into nine groups and incubated for 48 h with annexin V-FITC and PI and analyzed using flow cytometry. **(A)** Q4: In each panel the lower left quadrant shows cells that are negative for both PI and annexin V-FITC. Q3: Upper right quadrant shows annexin positive cells (early apoptotic). Q2: Upper left quadrant shows only PI positive cells, which are necrotic. Q1: Lower right quadrant shows annexin and PI positive cells (late apoptotic cells). (a) control group; (b) Ang II group; (c) XAV939 group; (d) FKN-KD group; (e) Ang II + FKN-KD group; (f) XAV939 + FKN-KD group; (g) Ex-FKN group; (h) Ang II + Ex-FKN group; (i) XAV939 + Ex-FKN group.**(B)** The rate of early apoptotic cells (Q3) is represented in a histogram. *p* value represents ^*^p < 0.05 compared with the control group;# *p* < 0.05 compared with Ang II group; ## *p* < 0.05 compared with XAV939 group; ### *p* < 0.05 compared with FKN-KD group; ** *p* < 0.05 compared with Ex-FKN group. Data are expressed as the means ± standard deviation (*n* = 3). Statistical analyses were performed using multi-way classification ANOVA.

To investigate the role of FKN in the early apoptosis of HK-2 cells, we tested nine groups of HK-2 cells treated for 48 h and then determined cell apoptosis by Annexin V-FITC/PI staining and flow cytometry analysis. As shown in Figure 2, the apoptosis rate was increased significantly in the FKN KD and XAV939 group compared with that of the controls, but was decreased significantly in the Ex-FKN and Ang II group. In addition, we found that the apoptosis rate was increased significantly in the Ang II+FKN-KD group compared to that of the Ang II group, but was decreased significantly in the XAV939+Ex-FKN group compared with that of the XAV939 group. Taken together, these data suggested an apoptotic effect upon downregulation of the *FKN* gene and an anti-apoptotic role of Ang II in HK-2 cells. Conversely, an anti-apoptotic role was observed upon upregulation of the *FKN* gene whereas XAV939 played an apoptotic role in HK-2 cells.

### FKN Mediates EMT via the Wnt/β-Catenin Signaling Pathway in HK-2 Cells

**Figure 3:**
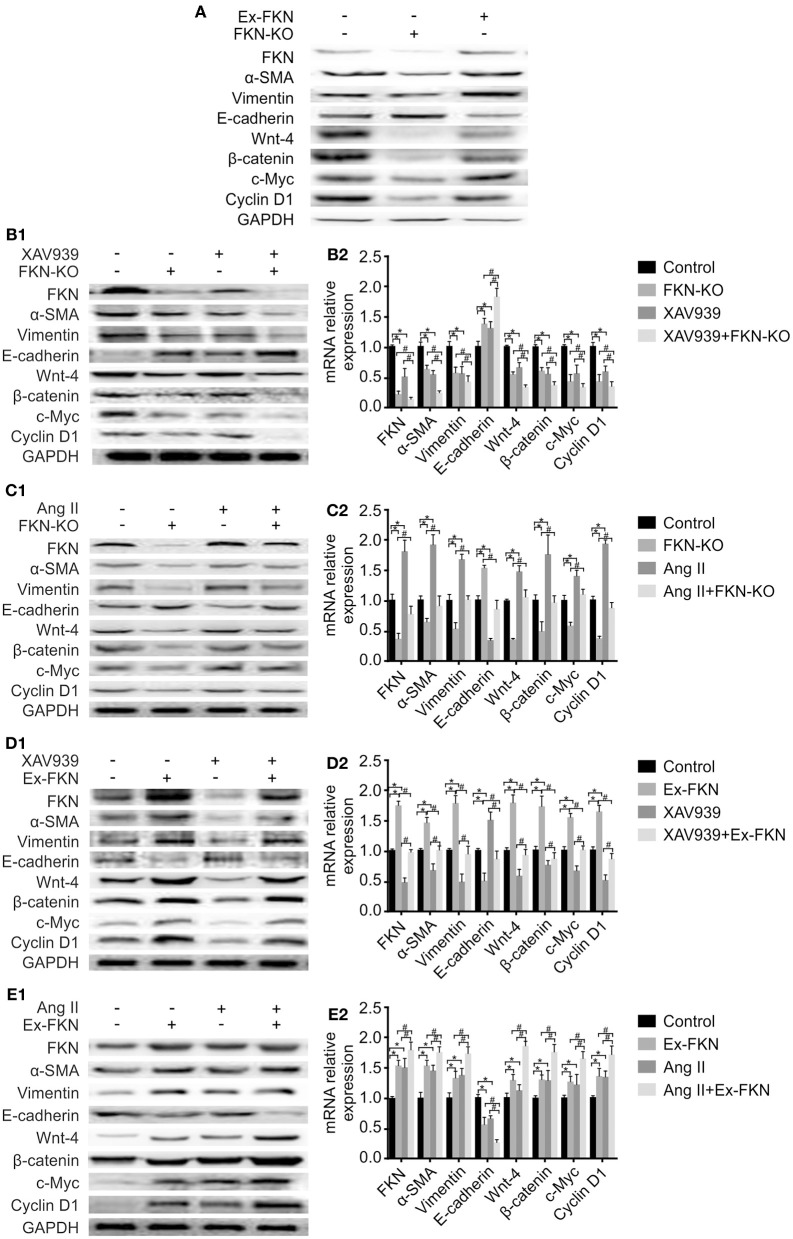
FKN participates in the EMT process of HK-2 cells via the Wnt/β-catenin signaling pathway. To detect the protein and mRNA levels of FKN, EMT markers (vimentin, α-SMA, E-cadherin) and Wnt/β-catenin pathway targets (Wnt-4, β-catenin, cyclinD1, and c-Myc) in different groups of HK-2 cells, cells were incubated for 48 h. Renal tissues extract (~50 μg) was resolved on SDS-PAGE and western blot analysis was performed using antibodies against FKN, vimentin, α-SMA, E-cadherin, Wnt-4, β-catenin, cyclinD1, and c-Myc. GAPDH was used as an internal control. Total RNA was extracted from renal tissue of mice. Then, the RNA was reverse-transcribed into cDNA and the transcripts were quantified using real-time PCR. *GAPDH* was used as an internal control. Data are expressed as the means ± standard deviation (*n* = 3). Statistical analyses were performed using one-way ANOVA. **(A)** Western blotting was used to detect the protein levels of FKN, vimentin, α-SMA, E-cadherin, Wnt-4, β-catenin, cyclinD1, and c-Myc in the FKN-KD group and Ex-FKN group. FKN-depleted HK-2 cells treated with 10 μmol/L XAV939 were assessed using western blotting **(B1)** and qRT-PCR **(B2)**. *p < 0.05 compared with the control group. #*p* < 0.05 compared with XAV939-treated FKN KD. FKN-depleted HK-2 cells treated with 10^−7^mol/L Ang II were measured using western blotting **(C1)** and qRT-PCR **(C2)**. *p < 0.05 compared with the control group. #*p* < 0.05 compared with Ang II-treated FKN KD. FKN-overexpressing HK-2 cells treated with 10 μmol/L XAV939 were assessed using western blotting **(D1)** and qRT-PCR **(D2)**. *p < 0.05 compared with the control group. #*p* < 0.05 compared with XAV939-treated FKN overexpression. FKN-overexpressing HK-2 cells treated with 10^−7^mol/L Ang II were assessed using western blotting **(E1)** and qRT-PCR **(E2)**.*p < 0.05 compared with the control group. #*p* < 0.05 compared with Ang II-treated FKN overexpression.

To further investigate the effect of FKN on EMT in HK-2 cells, we examined the expression of EMT-related proteins in treated cells through western blot analysis. As shown in Figure 3A, compared to the control group, KD of FKN in HK-2 cells inhibited the expression of the mesenchymal markers α-SMA and vimentin but stimulated the expression of the epithelial marker, E-cadherin, whereas FKN overexpression aggravated these. These results indicated that FKN overexpression could promote the process of EMT in HK-2 cells whereas FKN KD inhibits these. Owing to the important role of the Wnt/β-catenin signaling pathway in TIL and inflammation of LN, we then wondered whether FKN facilitated the HK-2 EMT process by activating Wnt/β-catenin signaling. To address this question, we examined the expression of Wnt-4, β-catenin, c-Myc, and cyclin D1, which are related to Wnt/β-catenin signaling. We found that FKN overexpression in HK-2 cells significantly increased the expression of Wnt-4, β-catenin, c-Myc, and cyclin D1. Conversely, KD of FKN in HK-2 cells suppressed the expression of these genes (Figure 3A). Furthermore, the hypothesis was further confirmed by treating HK-2 cells with the agonist Ang II and antagonist XAV939. As shown in Figures 3B1,C1,D1,E1, XAV939 significantly inhibited Wnt/β-catenin signaling as manifested by the reduced expression of Wnt-4, β-catenin, cyclinD1, and c-Myc, whereas Ang II activated this pathway in HK-2 cells. These results showed that the effect on EMT-related proteins was reversed by inhibiting the Wnt/β-catenin pathway in the FKN overexpression group, whereas it was promoted by activating the pathway in the FKN depletion group. Furthermore, consistent with the previous findings, addition of XAV939 in the FKN KD group further decreased the expression of EMT-related proteins, whereas these proteins were further increased by adding Ang II in the FKN overexpression group. The mRNA expression of E-cadherin and α-SMA as assessed by qRT-PCR showed similar results as those of the proteins, as shown in Figures 3B2,C2,D2,E2. Taken together, these results indicated that FKN mediates EMT through the Wnt/β-catenin signaling pathway in HK-2 cells.

### Effects of FKN on Renal Function and Serum Levels of ANA, Anti-dsDNA, and Anti-Sm in MRL/lpr mice

**Figure 4:**
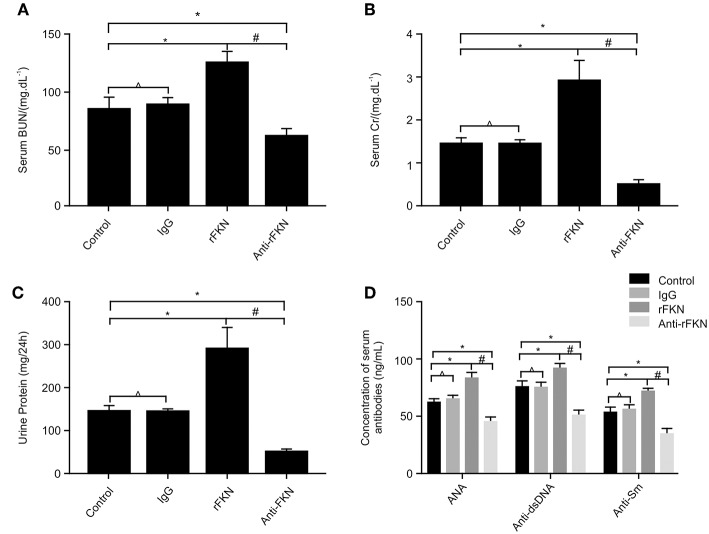
The effects of FKN on the levels of blood urea nitrogen, serum creatinine, 24 h urinary protein, ANA, anti-ds-DNA, and anti-ds-Sm determined at the end of 13 weeks. **(A)** Level of serum creatinine. **(B)** Level of blood urea nitrogen. **(C)** Level of 24 h urinary protein. **(D)** Level of ANA, anti-ds-DNA, and anti-ds-Sm. Control, MRL/lpr mice; IgG, MRL/lpr mice treated with isotype antibody; rFKN, MRL/lpr mice treated with recombinant-FKN antibody; anti-FKN, MRL/lpr mice treated with anti-FKN antibody. *p < 0.05 compared with the control group. No significant differences between IgG compared to the control group are indicated as *p > 0.05. Significant differences among the rFKN group and anti-FKN groups are indicated as #*p* < 0.05. Data are expressed as the means ± standard deviation (*n* = 3). Statistical analyses were performed using one-way ANOVA.

The production of numerous autoantibodies is a key feature of autoimmune disease, especially in LN, which plays an important role in the pathogenesis of kidney damage (40). To investigate the role of FKN on LN, at the end of the 12th week, MRL/lpr mice were IP injected with rFKN, anti-FKN, or IgG. We determined the expression levels of ANAs, anti-dsDNA, and anti-Sm antibody by ELISA in all study groups; in addition, serum Cr (SCr), BUN, and 24-h urinary protein (Upro) were also detected. As shown in Figures 4A–D, no difference was observed in the level of Scr, BUN, anti-dsDNA, ANA, anti-Sm, or Upro between the control and isotype group (*p* > 0.05), whereas the amount of BUN, Scr, anti-dsDNA, ANA, anti-Sm, and Upro was significantly increased in MRL/lpr mice after rFKN protein injection (*p* < 0.05). In comparison, treatment with anti-FKN antibody particularly decreased the amount of BUN, Scr, and Upro. These results suggested a significant role for FKN in the development of LN disease in MRL/lpr mice. The treatment with FKN antibody attenuated the progression of SLE in MRL-lpr mice as indicated by reduced levels of ANA, anti-dsDNA, and anti-SM antibodies in the plasma. The treatment also protected mice from progressive renal pathology as indicated by controlled renal parameters.

### FKN Involvement in EMT and TIL Relies on Wnt/β-Catenin Signaling in MRL/lpr Mice

**Figure 5:**
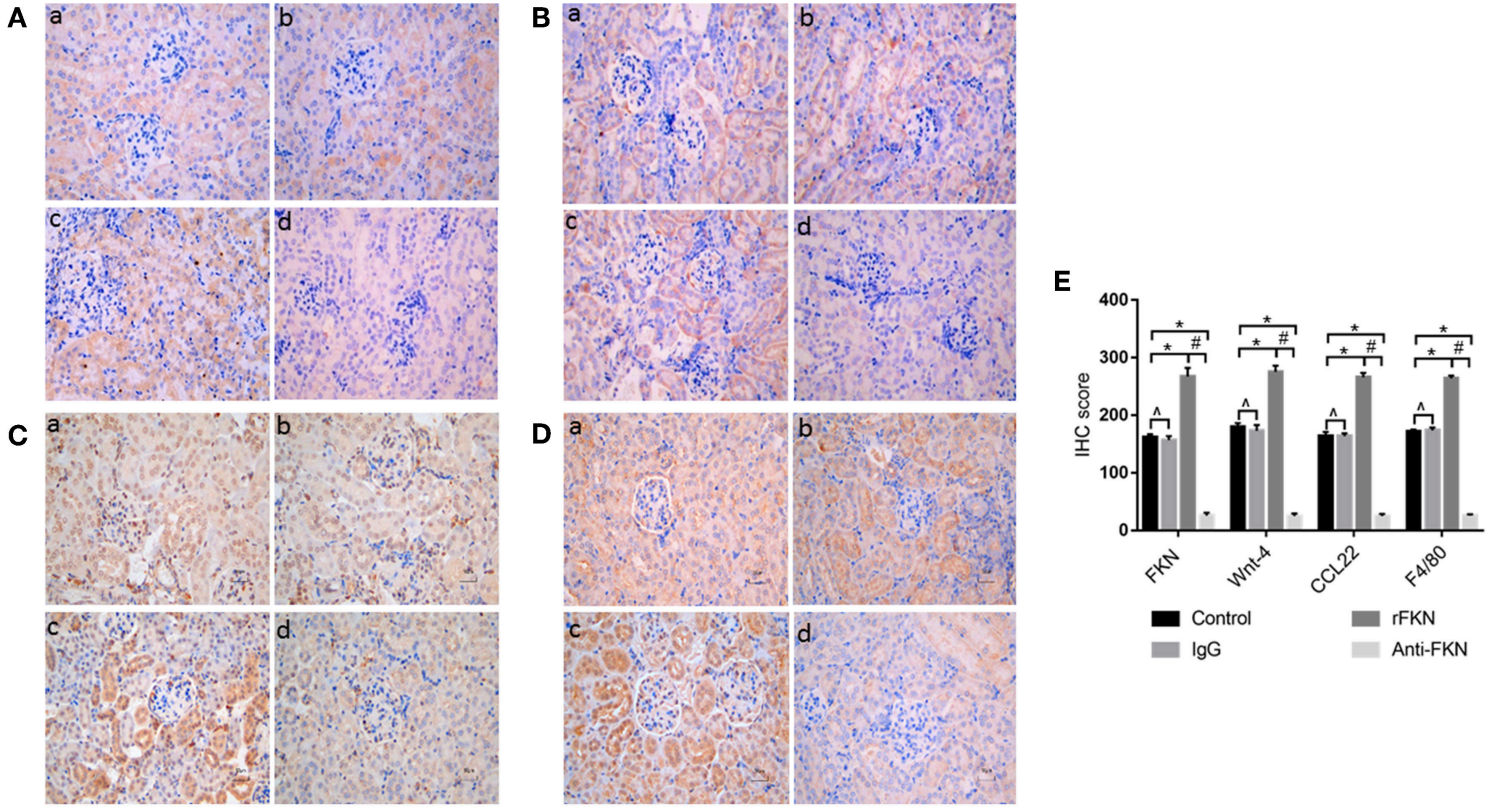
Twelve-week-old MRL/lpr mice were IP injected with isotype antibody, recombinant-FKN protein, and anti-FKN antibody for 7 days. In kidney sections of MRL/lpr mice stained by IHC (original magnification, ×400). (a) Control group; (b) IgG group; (c) rFKN group; (d) anti-FKN group. **(A)** Expression of FKN as examined by IHC staining in the renal tissues. **(B)** Expression of Wnt-4 as examined by IHC staining in the renal tissues. **(C)** Expression of CCL22 as examined by IHC staining in the renal tissues. **(D)** Expression of F4/80 as examined by IHC staining in the renal tissues. **(E)** The column diagram indicates the statistical of **(A-D)**. *p < 0.05 compared with the control group. No significant differences between IgG compared to the control group are indicated as *p > 0.05. Significant differences among the rFKN group and anti-FKN groups are indicated as #*p* < 0.05. Data are expressed as the means ± standard deviation (*n* = 3). Statistical analyses were performed using one-way ANOVA.

**Figure 6:**
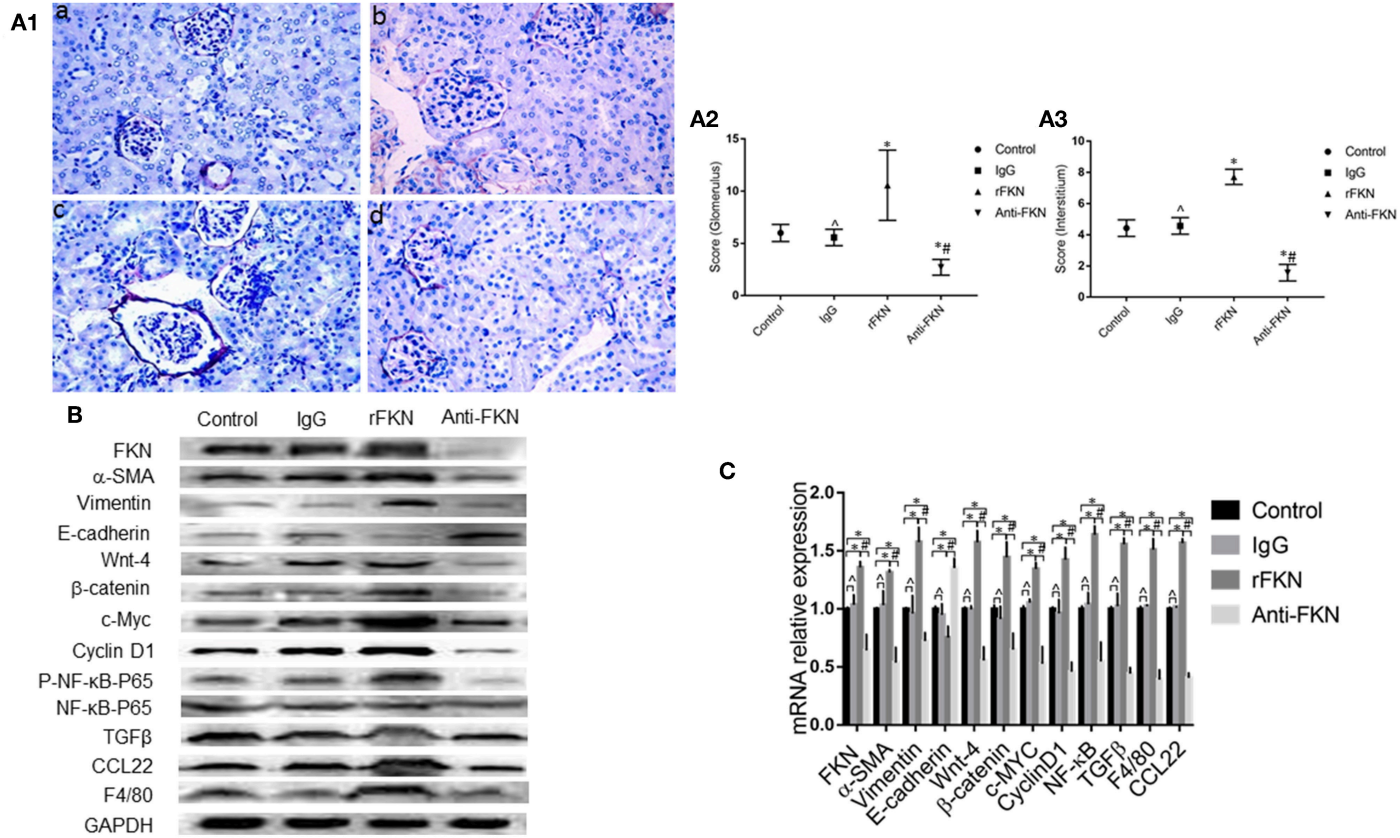
FKN is involved EMT and fibrosis via the Wnt/β-catenin pathway in the kidney of MRL/lpr mice. Histopathological features of renal tissue in the MRL/lpr mice model were studied by PASM staining (original magnification, ×400). (a) Control group;(b) IgG group; (c) rFKN group; (d) anti-FKN group. **(A1)** Assessment of glomerular and renal interstitial pathologies stained with PASM. Glomerular pathology was graded from the sum of scores for glomerular inflammation, thickness of basement membrane, epithelial cell reactivity, crescent formation, and necrosis. Interstitial pathology was graded using the sum of scores for perivascular inflammation and inflammatory cell infiltration. Scores were graded as 0 to 4 (0, none; 1 mild; 2 moderate; 3 moderate-high; 4 high). **(A2**) the sum of score by PASM staining in the renal glomerular inflammation. **(A3)** the sum of score by PASM staining in the renal glomerular inflammation. Renal tissue extract (~50 μg) was resolved on SDS-PAGE and western blot analysis was performed using antibodies against FKN, vimentin, α-SMA, E-cadherin, Wnt-4, β-catenin, cyclinD1, and c-Myc. GAPDH was used as an internal control. Total RNA were extracted from renal tissue of mice. Then the RNA was reverse-transcribed into cDNA and the transcripts were quantified using real-time PCR. GAPDH was used as an internal control. NF-kB-P65 was used as an internal control group of P- NF-kB-P65. Western blotting **(B)** and qRT-PCR **(C)** was used to detect the protein and mRNA levels of FKN, vimentin, α-SMA, E-cadherin, Wnt-4, β-catenin, cyclinD1, c-Myc, P- NF-kB -P65, CCL22 and F4/80 in renal tissues. *p < 0.05 compared with the control group. No significant differences between IgG compared to the control group are indicated as *p > 0.05. Significant differences among the rFKN group and anti-FKN groups are indicated as #*p* < 0.05. Data are expressed as the means ± standard deviation (*n* = 3). Statistical analyses were performed using one-way ANOVA.

To determine the role of FKN in EMT and whether this was associated with signaling via the Wnt/β-catenin pathway in the renal fibrosis of MRL/lpr mice, we detected the levels of EMT and Wnt/β-catenin pathway components with qRT-PCR and western blot assays in all study groups. We also examined the expression of FKN, wnt-4, CCL22, and F4/80 in the renal cortex by IHC. The expression of FKN, wnt-4, CCL22, and F4/80 protein was compared between the different groups of mice by IHC. The location of FKN, wnt-4, CCL22, and F4/80 protein were mainly seen in the cytoplasm of renal tubular epithelial cells, endothelial cells and some podocytes (Figures 5A–D). According to semiquantitative evaluation, the intensity scores of FKN, wnt-4, CCL22, and F4/80 were 162 ± 5, 157 ± 7, 267 ± 15, and 26 ± 5; and 180 ± 6, 173 ± 10, 275 ± 11 and 25 ± 4 and 164 ± 7, 163 ± 5, 265 ± 8, and 25 ± 4; 172 ± 3, 173 ± 5, 264 ± 5 and 26 ± 3 in the control, IgG, rFKN, and anti-FKN group mice, respectively. Furthermore, to identify whether FKN participates in tubulointerstitial fibrosis, we evaluated the renal sections stained with PASM and assessed by histological scoring for overall glomerular proliferative changes, crescent formation and necrosis, and interstitial inflammation. However, no crescent formation or necrosis was found. As shown in Figure 6A, histological sections from rFKN group mice exhibited more severe renal damage than those of the MRL/lpr group, including cellular proliferation, inflammation, glomerular expansion, mesangial proliferation, thickening of the mesangial basement membrane and interstitial inflammation. These changes are indicative of renal structural damage. MRL/lpr mice treated with anti-FKN presented significantly less renal damage that showed an abatement of the lowered mesangial expansion, glomerular inflammation and focal hypercellularity (reflected in the renal score, Figure 5E, *p* < 0.05). Conversely, no significant amelioration of the interstitial inflammation was observed in the IgG group. These results suggested that FKN is involved in kidney damage and contributes to accelerate renal fibrosis in MRL/lpr mice. In the renal cortex of the rFKN group, the expression of α-SMA and vimentin were significantly upregulated, whereas that of E-cadherin was obviously downregulated compared to that in the MRL/lpr control group. These results are consistent with a high level of Wnt/β-catenin signaling. Conversely, FKN and Wnt/β-catenin signaling were significantly decreased in the anti-FKN group, which led to the induction of a lower level of vimentin and α-SMA albeit a higher level of E-cadherin expression, as shown in Figure 6B. The mRNA expression of E-cadherin and α-SMA as assessed by qRT-PCR showed similar results as that of the protein (Figure 6C). The results indicated that rFKN group mice exhibited increased expression of glomerular FKN, wnt-4, CCL22, and F4/80 (*p* < 0.05) compared to that of MRL/lpr control group mice albeit decreased expression in mice treated with anti-FKN (*p* < 0.05). Furthermore, the involvements of NF-kB and TGFβ pathways in the regulation of EMT and TIL, and if it was due to the reduced accumulation of macrophages and macrophages-derived factors in renal tissue of mice treated with FKN antibody were demonstrated. The activation of NF-κB and expression of TGFβ, macrophages-derived factor CCL22 and macrophages marker F4/80 in renal cortex of MRL/lpr mice were evaluated by western blot and qRT-PCR. As shown in Figures 5B,C, recombinant-FKN stimulated the activation of NF-κB and upregulated the expression of TGFβ, CCL22, and F4/80, anti-FKN antibody suppressed the activation of NF-κB and downregulated the expression of TGFβ, CCL22, and F4/80 in MRL/lpr mice renal. These results suggested that the Wnt/β-catenin pathway accompanied by NF-κB and TGFβ pathway is involved in the FKN-mediated EMT and TIL, which is due to the accumulation of macrophages and macrophages-derived factors in the murine model of LN.

## Discussion

Accumulating evidence indicates that FKN is highly expressed in renal disease and is modulated by complicated regulatory systems (41). We previously found that in LN, FKN expression is significantly higher than that in peripheral blood mononuclear cells and serum from healthy controls. Notably, there is a positive correlation between FKN and the pathogenesis and activity of LN (42). In addition, our other findings showed that FKN expression levels were upregulated in HK-2 cells after stimulation with lipopolysaccharide and that as the level of FKN expression decreased, kidney function improved and kidney lesions decreased (32). However, little is known regarding the specific mechanism of FKN function in LN and no previous studies have, to our knowledge, investigated the role of FKN in EMT and TIL. In the present study, we firstly confirmed that the E-cadherin level was upregulated in FKN-depleted HK-2 cells, whereas those of E-cadherin, α-SMA, and vimentin were downregulated. In contrast, our findings indicated that overexpression of FKN in HK-2 cells enhanced EMT and might play an important role via an FKN-Wnt/β-catenin-EMT axis in HK-2 cells. In addition, we also examined the role of FKN in the development of TIL in LN mice. The results demonstrated that FKN facilitates TIL in LN mice. Specifically, anti-FKN antibody treatment could ameliorate TIL by improving EMT, inflammation, and fibrosis, suggesting that FKN could also be related to the progression of TIL *in vivo*. Moreover, our results suggested, for the first time, that FKN was involved EMT progression by activating Wnt/β-catenin signaling, which further enhanced the likelihood of LN leading to TIL.

The MRL/lpr mouse LN animal model was successfully cultivated at the Jackson Laboratory in the United States in the 1970s (43). The mouse presents lymphoproliferation (lpr), which is very similar to the clinical manifestations of human LN, including systemic lymph node and spleen enlargement, autoantibodies, excessive deposition of immune complexes, and imbalance of T lymphocyte subsets *in vivo* (44). Therefore, this model is widely employed in the research of LN. In the present study, we used the MRL/lpr mice as an animal model of LN and observed increased urine proteinuria, BUN, and Scr, in addition to apparent tubulointerstitial fibrosis, which suggested that MRL/lpr mice had developed LN at the 12th week.

The chemokine FKN constitutes a small, chemoattractant protein that recruits inflammatory cells at the site of inflammation (30). Accordingly, FKN is considered to represent a therapeutic target in chronic inflammatory disorders (45); however, increasing evidence suggests that FKN (CX3CL1) is more correlated with immune-related inflammatory diseases including experimental autoimmune encephalomyelitis (46) and rheumatoid arthritis (47), and is also expressed in macrophages, fibroblasts, endothelial, and dendritic cells (45). FKN binding to its unique receptor, CX3CR1, can promote hypertensive interstitial fibrosis in the kidney (48). In our previous studies, we have verified that FKN positively correlated with LN (42), especially in patients with active LN and those with renal damage (49). Other studies have also reported that FKN is involved in the development of glomerulopathy in humans, including crescentic glomerulonephritis (50). The role of FKN in renal injury associated with SLE has been studied and it indicated reduced migration of pro-inflammatory macrophages in FKN antibody treated mice is one of the major mechanisms for observed effect (51). The similar finding was observed in mouse model of renal fibrosis (52, 53). In the present study, upon IP injection of rFKN into MRL/lpr mice, we found that the kidney matrix of the mice was thickened and exhibited mesangial matrix breakage, the glomerulus was atrophied, the renal tubules were necrotic as shown by PASM staining, and decreased renal function was apparent as evidenced by increased urine protein. Conversely, after injection of an anti-FKN antibody, mesangial matrix thickening was alleviated and there was no rupture, glomerular atrophy was alleviated as shown by PASM staining, renal function recovery was determined, and urinary protein decreased. These results suggested that FKN levels are directly proportional to the degree of renal fibrosis and renal impairment, and suggested that FKN may constitute an important factor underlying the damage of renal fibrosis.

EMT plays an important role in the progression of TIL in human LN (54, 55). EMT is a pathologic process wherein epithelial cells transdifferentiate into motile mesenchymal cells after kidney damage. Considering the physiological characteristics of EMT, especially the role of renal tubular epithelial cells, we determined the influence of FKN on EMT ability and found that FKN clearly downregulated the expression of E-cadherin and upregulated the expression of α-SMA and vimentin. Moreover, the silencing of FKN could suppress the EMT process, which demonstrated that FKN represents a new target for modulating EMT. We therefore concluded that dysregulation of FKN could affect the natural processes of EMT.

To clarify the underlying mechanism by which FKN variation affected the EMT process, we noted that an increasing number of studies have reported that kidney damage might induce EMT via the Wnt/β-catenin pathway. As an important regulator of EMT, the Wnt/β-catenin pathway plays a crucial role in LN (56). Wang et al. also reported that the Wnt/β-catenin pathway was activated in LN and might play a role in renal fibrosis (18). Some scholars have proposed that the increase in Wnt pathway activation might lead to an increasing number of renal diseases in kidneys from lupus-prone NZB/NZW mice (57). The quiescent Wnt could be activated by inflammatory factors to promote β-catenin expression by escaping degradation and translocating into the nucleus. The binding of factor B to the LEF/TCF transcription factor further increases the expression levels of cyclin D1 and c-Myc, which lead to cell proliferation and differentiation disorders (58), in turn promoting the EMT ability of renal cells. Based on our findings that FKN is involved in the EMT process, we suggested that FKN might therefore promote the EMT process by activating the Wnt/β-catenin pathway. Consistent with this hypothesis, the results of the present study showed that high FKN expression enhanced the expression level of c-Myc and cyclin D1. To further confirm our view, we treated cell lines with XAV939 (a pathway inhibitor) or Ang II (a pathway activator). Our results demonstrated that XAV939 blocked and Ang II enhanced the EMT process induced by FKN, supporting our hypothesis. In view of previous studies reporting that FKN modulates the NF-κB signaling pathway, our study may thus suggest new directions for investigating the multiple functions of FKN. However, whether FKN can regulate NF-κB signaling in LN or whether there is cross-talk among FKN, NF-κB, and the Wnt/β-catenin pathway remains to be investigated.

We have embarked on further experimental studies on this question. As an important innate immune cell, macrophages can mediate the occurrence, and development of LN. On the one hand, as antigen-presenting cells, macrophages present antibodies to T cells by presenting autoantigens to the cells, resulting in LN; on the other hand, macrophages mediate LN by producing various pro-inflammatory mediators. At different stages of LN development, the microenvironment of the kidney changes, which makes the M1/M2 macrophage dynamically change and further affects the progression and prognosis of LN. M1 macrophages secrete TNF-α, IL-1β, IL-6, IL-23, chemokine ligand-9 (CXCL-9), CXCL-10, and other pro-inflammatory factors, synthesis loop Oxidase-2 (COX-2) and inducible nitric oxide synthase (iNOS) enhance antigen presentation and complement-mediated phagocytosis, thereby promoting inflammatory responses. M2 type macrophages are distinct from M1 type macrophages, and are classified into M2a, M2b, and M2c types according to their different functional phenotypes after activation. M2a cells are dependent on the activation of IL-4 and IL-13, secrete TGF-β1, synthesize arginase 1 and extracellular matrix, and are mainly involved in collagen production, tissue repair and wound healing, and promote Th2 immune response. Activation of M2b is mainly mediated by immune complexes and Toll-like receptors, secreting IL-10, inhibiting acute inflammatory responses caused by bacterial endotoxin, and promoting Th2 immune response; M2c is induced by IL-10 and glucocorticoids, secreting IL-10 and TGF-β1 mainly play a role in regulating and inhibiting inflammatory reactions (59). In contrast, exposure of intrinsic renal macrophages to apoptotic cells may promote an anti-inflammatory M2 phenotype associated with release of IL-10 and TGFβ and promotion of tubular repair; prolonged exposure to M2 macrophages may however result in renal fibrosis. Our results demonstrated that anti-FKN antibody treatment significantly inhibited expression of macrophages (F4/80) and macrophages-derived factors (CCL22) in renal tissue of mice, makes the opposite results after treatment with rFKN antibody. It is not surprising therefore, that macrophage function in injured kidneys may be quite variable. Pan et al. reported that most macrophages in the UUO model are biased toward F4/80^+^ CD163^+^ M2 type, and can secrete TGF-β in large amounts, enhance EMT-mediated renal fibrosis, and specifically clear M2 macrophages. Significantly reduce EMT-mediated renal fibrosis, reversal of M2 macrophages can aggravate renal fibrosis (60). After ischemia-reperfusion injury in lupus-susceptible mice, ischemia-induced tubular cells mediate the activation of M1 macrophages by producing a large number of colony-stimulating factors, aggravating repair dysfunction and inflammatory response, leading to the occurrence of LN (61), in the late stage of kidney injury, renal tubular hyperplasia repair, mainly in the M2 type macrophage infiltration in the kidney, promote the repair of epithelial cells and vascular endothelial cells, if the epithelial cells and blood vessels are not completely repaired, it will promote M2 type Polarization of macrophages leads to renal fibrosis. Finally, macrophages may have fibrolytic functions that although beneficial in repair of acute injury, may foster excessive remodeling, and damage in chronic nephritis (62).

NF-κB is a pleiotropic nuclear transcription factor that plays an important regulatory role in the pathogenesis of LN. In the renal lesions of LN patients and lupus mice, NF-κB pathway activation was found; at the same time, downstream factors regulated by the NF-κB classical pathway, such as TNF-α, IL-1β, IL-6, and intercellular adhesion molecules—The expression of 1 (ICAM-1) and the like also increased significantly. By activating the NF-κB pathway, HMGB1 regulates the transcription of the cell proliferation-associated protein D1 and promotes the proliferation of mesangial cells, thereby participating in the pathogenesis of LN (63). The degree of activation of the NF-κB pathway in the glomerulus is related to the SLE activity index and macrophage infiltration. Studies have (64) found that NF-κBp65/p50 promotes the polarization of M1 macrophages. Mesenchymal stem cells have anti-inflammatory and immunosuppressive effects. After 28 weeks of injection of mesenchymal stem cells in the tail vein of lupus mice, the renal inflammatory response is relieved. This effect is mainly through increasing the number and phagocytic capacity of M2 macrophages (65). Bone marrow-derived mesenchymal stem cells promote M2-type macrophage polarization, which is associated with inhibition of NF-κB pathway by bone marrow-derived mesenchymal stem cells (66). Our results demonstrated that anti-FKN antibody treatment significantly inhibited expression of NF-κB p65 and TGFβ, the 24h urine protein level and serum anti-double-stranded DNA antibody level of lupus mice were significantly decreased, and the degree of renal pathological inflammatory reaction was reduced. These effects are likely to be associated with reduced accumulation of macrophages (F4/80) and macrophages-derived factors (CCL22) in renal tissue of mice, makes the opposite results after treatment with rFKN antibody. The data presented here are consistent with previous observations effects of NF-κB inhibitors resulting in a reduced FKN expression in MRL/lpr mice (67). Due to the plasticity of macrophages, not only untyped macrophages will be polarized to other types, but also differentiated M1/M2 macrophages will be transformed by the microenvironment. The dynamic balance of M1/M2 macrophages is disrupted at different stages of LN and is closely related to the prognosis of LN, which is regulated by a variety of signaling pathways. We suggest that more studies are needed to explore the underline mechanism and therapeutic strategy of the role of FKN in patients with LN.

In summary, our results revealed, for the first time, evidence for the existence of an FKN-Wnt/β-catenin-EMT axis that promotes the EMT capability and TIL process in the kidneys of MRL/lpr mice and HK-2 cells. A better understanding of the multiple functions of FKN may provide new guidance for developing targeted therapies to treat LN. Specifically, although further investigation is needed, findings strongly suggest that FKN may serve as a potential therapeutic target and prognostic biomarker against LN in the future.

## Ethics Statement

The study was approved by the Committee of Animal Care and Use of Youjiang Medical University for Nationalities, and all procedures were performed according to the National Institutes of Health Guidelines.

## Author Contributions

DF, SS, and JW carried out the experimental work. YY and DF participated in the design of the study and together performed the statistical analysis. YY helped to draft the manuscript. All authors read and approved the final manuscript.

### Conflict of Interest Statement

The authors declare that the research was conducted in the absence of any commercial or financial relationships that could be construed as a potential conflict of interest.
